# Inhibitory Effect of Gardenoside on Free Fatty Acid-Induced Steatosis in HepG2 Hepatocytes

**DOI:** 10.3390/ijms161126058

**Published:** 2015-11-20

**Authors:** Huiqing Liang, Limin Zhang, Hongguo Wang, Jinmo Tang, Jiaen Yang, Chuncheng Wu, Shaodong Chen

**Affiliations:** 1 Department of Traditional Chinese Medicine, Medical College of Xiamen University, 422 S. Siming RD, Xiamen 361005, China; 13850005898@163.com (H.L.); 124520141153580@stu.xmu.edu.cn (L.Z.); 24520131153546@stu.xmu.edu.cn (H.W.); 2 Xiamen Hospital of Traditional Chinese Medicine, 1739 Xianyue RD, Xiamen 361009, China; 13850085858@163.com (J.T.); yje1977@yeah.net (J.Y.); wccxmtcm@yeah.net (C.W.)

**Keywords:** gardenoside, free fatty acid, HepG2, steatosis, inflammatory cytokines, NFκB

## Abstract

Gardenoside is one of the most important effective extractions of a herb for its hepatoprotective properties. The aim of this study was to address the mechanism of Gardenoside on HepG2 cellular steatosis induced by free fatty acids (FFAs). The model of HepG2 steatosis was duplicated by oleic and palmitic acid at the proportion of 2:1 (FFAs mixture) for 24 h, then lipid toxicity was induced. 3-(4,5-Dimethylthiazol-2-yl)-2,5-diphenyltetrazolium bromide (MTT) were used to detect cell viability and Oil Red O staining method was used to judge the lipid accumulation respectively. Inflammatory cytokines TNF-α, IL-1β, IL-6 and intracellular NFκB were measured after 24 h. The steatosis was significantly decreased after Gardenoside treatment without cytotoxicity. TNF-α, IL-1β, IL-6 were modulated to HepG2 cells by treatment of Gardenoside. In the meantime, the activation of NFκB was inhibited by Gardenoside. Gardenoside has a protective effect on FFA-induced cellular steatosis in HepG2 cells which indicates that Gardenoside might be a potential therapeutic herb against NASH by suppressed supernatant inflammatory cytokine production and intracellular NFkB activity.

## 1. Background

Non-alcoholic fatty liver disease (NAFLD) has four stages: simple steatosis, non-alcoholic steatohepatitis (NASH), fibrosis and liver cirrhosis, which cannot be explained by alcohol consumption [1,2].

As we know, the “two-hit hypothesis” is the most important pathogenesis of NAFLD [3]. The first hit is the development of hepatic steatosis via accumulation of triglycerides in hepatocytes, and the “second hit” involves hepatic injury, inflammation, which are closely associated with oxidative stress in the liver [4,5,6]. To study hepatic steatosis *in vitro*, a cellular hepatic model was previously established by treating human HepG2 cells with free fatty acids (FFAs) [7]. In the human body, the main fatty acids include the palmitic acid (PA) and oleic acid (OA) [8]. PA and OA are widely used to induce steatosis *in vitro* [9].

The goal of managing NAFLD is to treat steatosis. To date, no standard treatment is recognized; the most efficacious treatment for NAFLD is represented by an adjusted diet and physical exercise. It was reported that increased physical activity and improvements in diet are good for weight management to treat NAFLD. Therefore, lifestyle modification should be the first step for NAFLD patients when developing a treatment plan for them [10].

It is reported that Traditional Chinese Medicine (TCM) plays an important role in treating NAFLD [11]. However, the mechanisms of TCM for treating NAFLD still remain somewhat unclear. Recently, extraction from herbs is increasingly becoming the effective way to solve the problems of TCM [12]. Gardenoside is the main effective extraction of Gardenia jasminoides Ellis, which is an indigenous medicinal herb widely used for hepatoprotective, analgesic, and antipyretic drug [13]. As is known, obesity and NAFLD have been major public health problems; relevant approaches to the therapeutic activities of Gardenoside are noteworthy. In our previous study, Gardenoside was applied for treatment on NASH [14,15]. However, the mechanism is still somewhat unclear.

In this study, the hepaticlipotoxicity model of HepG2 cell steatosis and TNF-α secretion induced with FFAs, which has been reported by Feldstein [15], was duplicated to observe the effect and mechanism of Gardenoside on NASH.

## 2. Results

### 2.1. Effects of Gardenoside on Cell Viability

**Figure 1:**
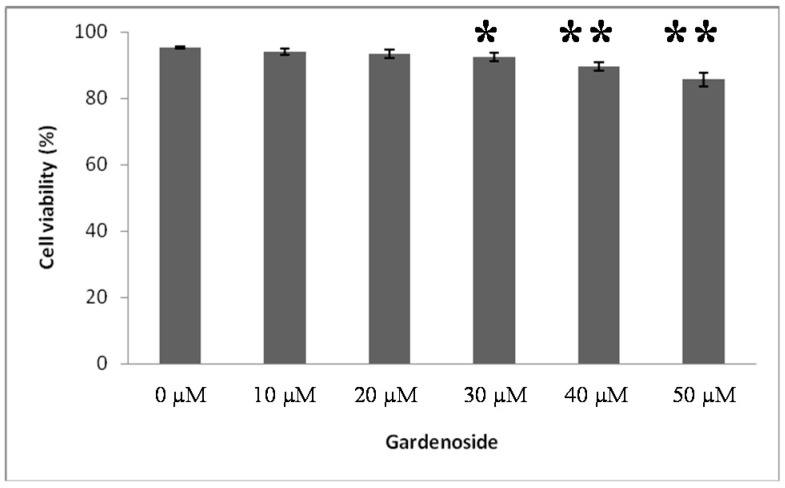
Cell viability assay. After treatment of Gardenoside and free fatty acids (FFAs) on the HepG2 cells, MTT assay was performed. Gardenoside was treated as 0, 10, 20, 30, 40 and 50 µM for 24 h. 10 and 20 µM of Gardenoside showed no toxicity to HepG2 cells. All experiments were repeated at least three times. * *p* < 0.05, ** *p* < 0.01, compared to 0 µM Gardenoside group.

To determine whether the treatment of Gardenoside on HepG2 cells has value for medical use with no toxic effect, the cells were treated with different concentrations of Gardenoside (10, 20, 30, 40 and 50 µM) for 24 h and cell viability was evaluated by MTT assay. Quantities of 30, 40 and 50 µM of Gardenoside were significantly toxic to HepG2 cells (*p* < 0.05, *p* < 0.01). In contrast, 10 and 20 µM of Gardenoside showed no substantial decrease in cell viability, which were used for further studies (Figure 1).

### 2.2. Effect of Gardenoside on Steatosis

**Figure 2:**
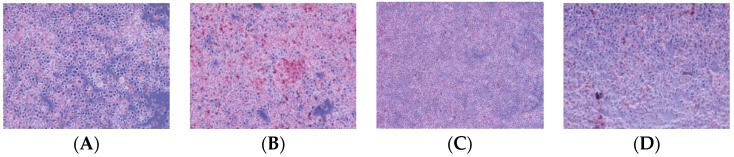
Oil Red O staining. Lipid accumulation in HepG2 cells induced by FFAs for 24 h. Lipid droplets in HepG2 cells was observed by microscope (400×): the control cells treated with only 5% BSA (**A**); cells treated with FFAs for 24 h (**B**); cells pretreated with FFAs for 24 h and cultured with Gardenoside 10 µM for 24 h (**C**); cells pretreated with FFAs for 24 h and cultured with Gardenoside 20 µM for 24 h (**D**).

HepG2 cells were incubated in the mixture with PA and OA for 24 h, which can lead to steatogenesis simultaneously in hepatocytes. Then cells were stained with Oil Red O solution for 30 min, and the increased intracellular lipid contents dyed pink were visually observed by microscope (400×) (Figure 2A,B). Gardenoside can decrease the lipid droplets in HepG2 cells cultured with FFAs (Figure 2C,D).

**Figure 3:**
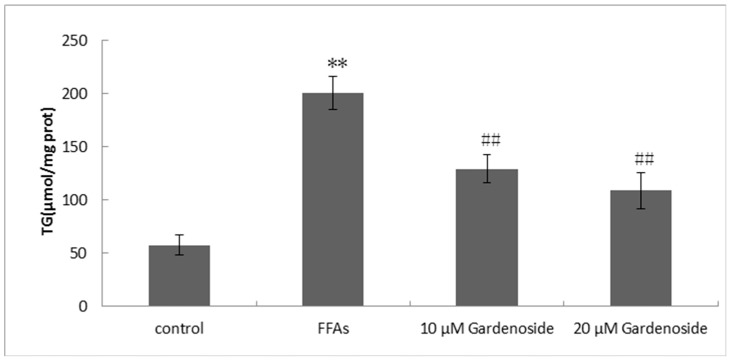
Effect of Gardenoside on triacylglycerol accumulation in FFAs induced hepatic steatosis in HepG2 cells. The Triglyceride (TG) level of the cells was measured by using TG assay kit. All experiments were repeated at least three times. ** *p* < 0.01, compared to control group, and ## *p* < 0.01, compared to FFAs group.

The HepG2 cells were treated with 0.5 mM concentration of FFAs for 24 h to induce hepatic steatosis. Cells treated with 5% BSA were used as control. TG was accumulated as lipid droplets in the FFAs treated cells and Gardenoside significantly decreased TG content by 36% (10 µM), and 46% (20 µM), as given in Figure 3.

### 2.3. Changes of Inflammatory Cytokines in Supernatant

Chronic hepatic inflammation is closely associated with the pathogenesis of NAFLD. In fact, lipid peroxidation increases the production of inflammatory cytokines and activates increased levels of ROS, leading to oxidative stress. FFAs increased TNF-α, IL-6, IL-1β by 4.44-, 4.98-, 5.78-fold compared to control cells. Gardenoside significantly reverted the decreased TNF-α by 45.87% (10 µM), 51.19% (20 µM), IL-6 by 24.05% (10 µM), 43.28% (20 µM), IL-1β by 45.22% (10 µM), 46.47% (20 µM), compared to FFAs group in Table 1.

**Table 1:** Effect of Gardenoside on inflammatory cytokines in HepG2.

Groups	TNF-α (pg/mL)	IL-6 (pg/mL)	IL-1β (pg/mL)
Control	23.32 ± 1.86	19.16 ± 1.15	15.76 ± 2.04
FFAs	102.71 ± 6.89 **	95.52 ± 10.65 **	91.16 ± 6.93 **
10 µM Gardenoside	55.62 ± 6.74 ^##^	72.53 ± 7.71 ^#^	49.93 ± 5.32 ^##^
20 µM Gardenoside	50.13 ± 5.65 ^##^	54.16 ± 7.84 ^##^	48.82 ± 4.25 ^##^

Effect of Gardenoside on inflammatory cytokines in HepG2. To determine the effect of Gardenoside on inflammatory cytokines, HepG2 cells were treated with Gardenoside in the presence of FFAs. The production and secretion of inflammatory cytokines TNFα, IL-6 and IL-1β were significantly increased after FFAs treatment. However, Gardenoside treatment at all 10 or 20 µM concentrations decreased the expression of these inflammatory cytokines. Three independent experiments were carried out in triplicate. ** *p* < 0.01, compared to control group, and ^#^ *p* < 0.05,^##^ *p* < 0.01, compared to FFAs group.

### 2.4. Changes of Phospho-NFkB Protein Expression

**Figure 4:**
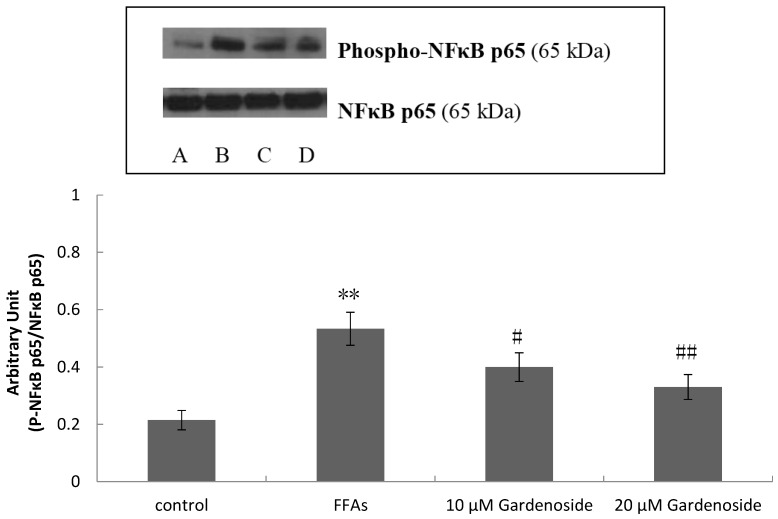
Changes of Phospho-NFκB p65 Protein Expression. NFκB p65 and Phospho-NFκB p65 protein expression was assessed using immunoblot analysis in HepG2 cells after incubation with or without FFAs in the presence or absence of Gardenoside 10 or 20 µM. HepG2 cells treated with DMSO were used as control (**A**), cells were induced by FFAs (**B**); cells were treated with Gardenoside 10 µM (**C**); 20 µM (**D**) for 24 h. Immunoblots are representative of three different experiments. ** *p* < 0.01, compared to control group, and # *p* < 0.05, ## *p* < 0.01, compared to FFAs group.

HepG2 cells treated with DMSO were used as control (A), cells were induced by FFAs (B), cells were treated with Gardenoside 10 µM (C), 20 µM (D) for 24 h. FFAs indicates the activity of Phospho**-**NFkB p65 in HepG2 cells. In contrast, this was reverted by Gardenoside as shown in Figure 4.

## 3. Methods

### 3.1. Cell Line and Culture

Human HepG2 cell line (purchased from the cell line bank, Shanghai Institute of cell biology, Chinese Academy of Sciences, Shanghai, China) was maintained in Hyclone Dulbecco’s Modified Eagle’s Medium (DMEM; Thermo Scientific, Rockford, IL, USA) supplemented with 10% heat-inactivated Fetal Bovine Serum (FBS, Atlanta Biologics, GA, USA), 2 mM l-glutamine, 100 U/mL penicillin, 100 μg/mL streptomycin, and 25 mM Hepes (all from Life Technologies, CA, USA). Cells were cultured in 5% CO_2_ at 37 °C.

### 3.2. Modeling

After reaching 80% confluence, the HepG2 cells were cultured with fetal bovine serum-free medium containing 5% bovine serum albumin (BSA) overnight in a 6-well plate. Sodium PA and OA (Sigma, St. Louis, MO, USA) were conjugated to FFAs (OA:PA at 2:1). The concentration of 50 mM stock solution of FFAs was dissolved in isopropanol. Final concentration of the FFAs was 500 μM. HepG2 cells were treated with 200 μL of 500 μM FFAs or with different concentrations of Gardenoside for 24 h. HepG2 cells cultured in a medium containing 5% BSA were used as a control [16].

### 3.3. Gardenoside Treatment

Gardenoside (Purity ≥ 98%, purchased from Zelang Medical Technology Co., Ltd., Nanjing, China) came from Gardenia jasminoides Ellis, which was dissolved in double-distilled H_2_O (ddH_2_O), diluted in DMEM, and used to treat HepG2 cells. The final quantity of solvent did not exceed 0.1% of culture media for all experiments.

### 3.4. Cell Cytotoxicity Detection

HepG2 cells were plated in 96-multiwell culture plates at 1 × 10^5^ cells per well. To study cytotoxicity, 24 h after plating, the medium was discarded and fresh medium containing 0, 10, 20, 30, 40 and 50 µM Gardenoside were added. After 24 h of incubation, cell viability was determined by colorimetry using the 3-(4,5-Dimethylthiazol-2-yl)-2,5-diphenyltetrazolium bromide (MTT) (USB corporation, Cleveland, OH, USA; Sigma-Aldrich, Oakville, ON, Canada). Insoluble formazan crystals were dissolved in DMSO and measured at 570 nm with a Bio-Rad model 680 microplate reader (Bio-Rad, Hercules, CA, USA).

### 3.5. Triglyceride Colorimetric Assay (TG Assay)

To determine intracellular TG level, HepG2 cells were plated in 6-well plates at 1 × 10^6^ cells per well, 10 and 20 µM Gardenoside and 500 μM FFAs were added at the same time for 24 h. The TG level of the cells was measured by using TG assay kit (Dongou Bioengineering Co., Ltd., Wenzhou, China). The cell lysate protein concentrations were measured using the BCA method.

### 3.6. Oil Red O Staining

HepG2 cells were washed three times with PBS, and then fixed with 10% formalin for 1 h. Cells were washed with 60% isopropanol briefly and incubated with 60% filtered Oil Red O solution (0.7 g per 200 mL of isopropanol, which was purchased from Sigma Chemical, St. Louis, MO, USA) for 30 min at room temperature after washing three times with distilled water. Then, cells were stained with hematoxylin (Sigma-Aldrich). Cells with Oil Red O Staining were observed under a microscope (Nikon, Tokyo, Japan).

### 3.7. Cytokine Assay

Samples of supernatant of HepG2 cells were collected after incubated with Gardenoside and FFAs for 24 h. The anti-inflammatory cytokines TNF-α, IL-6 and IL-1β were measured by enzyme linked immunosorbant assay (ELISA) kits (Biolegend Inc., San Diego, CA, USA).

### 3.8. Western Blot Analysis

Proteins were extracted from the cells using extraction buffer, which include 50 mM Tris HCl, 0.5% sodium deoxycholate, pH 8.0, 1% Nonidet P-40, 5 mM EDTA, 0.1% SDS, 150 mM NaCl, 1 mM PMSF, 1 mM NaVO_4_, 1 mM NaF, and protease inhibitor cocktail (Roche, Mannheim, Germany). Protein extracts were separated on 10% polyacrylamide gels and electrophoretically transferred onto polyvinylidene fluoride membrane (Gelman Laboratory, Ann Arbor, MI, USA). After blocking, the membranes were incubated with a primary antibody, and then with horseradish peroxidase-conjugated IgG (Santa Cruz Biotechnology, Santa Cruz, CA, USA). Blots were developed using ECL Detection Kit (Amersharm Pharmacia, UK). Primary antibodies to Phospho-NFkB p65, NFkB p65 were from Santa Cruz Biotechnology (Santa Cruz, CA, USA) and β-actin was from Sigma (St. Louis, MO, USA). Results of western blotting were normalized with those obtained for β-actin. Protein bands were quantified using densitometry with accompanying software (ImageJ).

### 3.9. Data Analysis

All data represent at least three separate experiments and each experiment was performed in triplicate. The significance of the data was analyzed with Prism 5 software (GraphPad Software, La Jolla, CA, USA) with one-way ANOVA and Bonferroni’s post-hoc test to compare each set of data. Bars show the means ± SEMs. The differences between the means were considered significant when *p* < 0.05.

## 4. Discussion

FFAs lead to an obvious accumulation of lipid droplet in the cytoplasm. Accumulation of lipid droplets in cells can be toxic manifesting in apoptosis if high content are reached [17,18]. Some investigations have been carried out in order to address FFAs’ induced lip toxicity in cells and some interest has been developed showing insight toward lipid accumulation-induced lip toxicity in cells [19]. In the present study, we found that FFAs increased TG content and lipid droplets in cells indicating the lipid accumulation and liptoxicity in HepG2 cells by FFAs. We confirmed that Gardenoside has function to decrease the intracellular TG content.

NFκB is an important regulator in inflammatory processes: NFκB activation has a close relationship to obesity and insulin resistance in different diseases [20]. FFAs can boost NFκB activity on cell surface receptor binding [21]. These ligand-receptor interactions trigger the recruitment of adaptor proteins and receptor-proximal kinases intimately culminating in the activation of the inhibitor of NFκB (IκB) kinase (IKK) complex. IKK subsequently phosphorylates IκB, which is then downgraded by the proteasome. Decreased levels of IκB free NFκB, which enable cytosolic-nuclear translocation and ultimately transcriptional induction of a large amount of genes involved in immune function [22]. The NFκB family contains five members including RelA(p65), RelB, c-Rel, p50/p105 (NFκB1) and p52/p100 (NFκB2), which form multifarious combinations of homodimers or heterodimers. p65/p50 is also called “classical” NFκB activation [23]. Upon stimulation, via a phosphorylation-dependent proteasome-mediated mechanism, IκB is degraded and the released NFκB is translocated to the nucleus where it binds to the κB-sites and regulates the transcription of target genes. This study shows that Gardenoside inhibits expression of Phospho-NFκB p65 protein, suggesting the drug has function to inhibit the NFκB activation.

TNF-α is one of the most important pro-inflammatory cytokine, which involved in systemic inflammation and is a member of cytokine family that stimulates acute inflammation. Various types of cells in the body produce TNF-α. TNF-α is also major mediator in various physiological processes, such as inflammation and cell proliferation [24]. It is reported that TNF-α is the most critical inflammatory cytokine in the progression of fatty liver [25]. IL-1β is another potent proinflammatory cytokine [26]. It was found that the levels of pro IL-1β increased significantly in the liver and in the serum of patient with NAFLD [27,28]. As inactive pro-IL-1β in response to inflammatory stimuli, IL-1β is produced, including both microbial products and endogenous danger-associated molecules. The role of IL-6 in NAFLD is complex and not well understood. The previous opinion is that when IL-6 released into the circulation, it was taken up by the liver, and then the resident hepatocytes regarded the IL-6 as a stimulus to begin production of acute phase proteins. Similarly, a principal hypothesis posted that *in situ* ations where an injury is inflicted directly upon the liver, it is the resident immune cells, such as the Kupffer cells (hepatic macrophages), that primarily produce the IL-6 used for stimulating acute phase protein production [29,30,31]. In both acute and chronic inflammation, NFκB is considered a key factor in regulating the immune response to infection. Activation of NFκB can induce the expression of TNF-α, IL-1β and IL-6, which increases the expression of proinflammatory molecules [32,33]. The study showed that in non-cytotoxic dose range (10 and 20 µM), Gardenoside does well in decreasing inflammatory cytokines secretion, such as TNF-α, IL-1β and IL-6. In the mean time, the expression of NFκB p65 activation also was inhibited by Gardenoside. These results suggest that Gardenoside has a direct, high-intensity intervention effect for hepatic lip toxicity.

## 5. Conclusions

NAFLD is one of the most common liver diseases. Excessive FFAs are harmful to the liver, which will induce over oxidative stress, inflammatory cytokine, hepatic steatosis and liptoxicity, and then contribute to disease progression. In this study, we provided evidence that Gardenoside has a protective effect on FFA-induced cellular steatosis in HepG2 cells. Gardenoside significantly suppressed supernatant inflammatory cytokine production and intracellular Phospho-NFkB p65 activity. Our results demonstrated that the effect mechanism of Gardenoside inhibits the hepatic liptoxicity and has a close relationship with inhibition on inflammatory cytokine secretion.
